# Medical Colleges and Quackery

**Published:** 1882-07-20

**Authors:** 


### EDITORIALS AND MISCELLANEOUS.

### "*MEDICAL COLLEGES AND QUACKERY.*"

Under this head the editor of the Atlanta Medical Register, in the July number, after some very timely and sensible comments touching the duties of Medical Colleges in properly and thoroughly training the students under their care, and the just expectation of the profession and the public that only correct principles be inculcated and enjoined upon those receiving diplomas, thus remarks:

" Having once felt these impulses, and being still impressed with the value of such influences, our astonishment can be imagined when, only a few days ago, we discovered in the list of recent graduates of an old and well-known Medical College the name of a notorious nostrum vendor. Standing side by side with aspiring and worthy young men, he received the indorsement of a corps of dignified professors and was sent out to the world with the seal of the institution upon him, not to practice medicine, ah, no; not that, no such drudgery for him; but, under cover of a diploma, unconditionally granted, to continue to ply his vocation undisturbed, and to continue to dispense his secret compound without the fear of the law before his eyes.

We are not apprised of the arguments advanced by these gentlemen, if any there were, to explain or excuse this act. We are merely dealing with the fact as it appears in their printed announcement and with the principle which the act involves, and whatever motive may have prompted them to lend their aid to the accomplishment of this unhallowed end, we emphatically assert that it was not considerate of the class; it was not complimentary to the alumni; it can scarcely reflect credit upon the College, and it surely will not tend to exalt the standing of the medical profession.

As the name of the Institution against which the above charge is brought is not given, we feel it just and proper to inform the profession that it does not apply to the Southern Medical College of this city, with which two of our editorial corps are connected; and we take occasion to say in behalf of the Faculty of this Institution (the new school) that no "notorious nostrum vendors" can be found among their list of graduates; and we take occasion to repeat, what has often been avowed by the founders of the Southern Medical College, that they have earnestly and dilligently labored to elevate the standard of medical education in the South, and that it is their aim to establish a great leading, central Institution in Atlanta for the Southern States; one of which the profession will be proud, and whose diploma will convey to its possessor a title to high and honorable recognition in the ranks of the medical profession everywhere.

It is hoped that the friends of medical education will appreciate these pledges, and will give to the new Institution their hearty co-operation and support.

